# Appearance of Maxwell’s spot in images rendered using a cyan primary

**DOI:** 10.1016/j.visres.2019.10.004

**Published:** 2019-12

**Authors:** Marina Gardasevic, Robert J. Lucas, Annette E. Allen

**Affiliations:** Division of Neuroscience and Experimental Psychology, Faculty of Biology, Medicine and Health, University of Manchester, M13 9PT, UK

**Keywords:** Maxwell’s spot, Metamerism, Multiprimary displays, Melanopsin, Colour vision, Macular pigment, CIE, Commission Internationale de ĺEclairage, LMS, long-, medium- and short-wavelength cones, RGB, red, green, blue, VCGYR, violet, cyan, green, yellow, red

## Abstract

The discovery of melanopsin as a third type of retinal photoreceptor, contributing to both perceptual vision and reflex light responses, represents a new opportunity to optimise the design of artificial light sources for practical applications and to generate experimental stimuli. In the case of emissive displays, multiprimary designs incorporating a cyan primary could be used to allow melanopic radiance to be controlled independent of colour and luminance. Here we explore the performance a five-primary (violet, cyan, green, yellow, red) display device and find an anomaly in colour appearance when the cyan primary is employed. The anomaly took the form of a reddish/pinkish tinge in the central visual field, consistent with descriptions of Maxwell’s spot. This effect was apparent in some full colour images and in uniform discs over a range of chromaticities. Its appearance in coloured discs correlated with differences in calculated colour coordinate between central and peripheral vision. A simulation indicated that inclusion of any primary with predominant output in the 470–500 nm range has the potential to produce such a discrepancy in central vs peripheral appearance. Applying an additional constraint in colour processing to reproduce naturally occurring differences in central vs peripheral colour coordinate eliminated appearance of the spot and produced acceptable colour images.

## 1 Introduction

Multi-primary displays that render images in more than the standard 3 distinct colour emitting elements (red, green, blue; RGB) can offer advantages in terms of energy efficiency and colour gamut (Teragawa, 2012). They can also provide an opportunity to control the activity of the inner retinal photoreceptor melanopsin. Melanopsin renders certain retinal ganglion cells photosensitive (Provencio et al., 2000, Berson et al., 2002, Lucas et al., 2003). These photosensitive retinal ganglion cells have been appreciated as the main input into the circadian clock, providing a signal of ambient irradiance that synchronises the clock’s phase with the solar day (Berson et al., 2002, Do and Yau, 2010, Schmidt et al., 2011). More recently, melanopsin has been implicated in ‘image-forming’ vision, both in studies with mice and with humans (Hankins and Lucas, 2002, Zaidi et al., 2007, Schmidt et al., 2014, Spitschan et al., 2017, Allen et al., 2017). In humans, melanopsin has been shown to have a behavioural significance in brightness discrimination and pattern detection (Allen et al., 2019, Brown et al., 2012, Spitschan et al., 2017, Yamakawa et al., 2019, Zele et al., 2018b).

Regulating melanopsin with artificial lighting and displays is desirable for a number of reasons, including modulating the circadian clock and adjusting perceived brightness. Melanopsin is maximally sensitive in the ‘cyan’ portion of the visible spectrum (around 480 nm (Bailes & Lucas, 2013)), and including an additional primary with dominant emission over these wavelengths can allow effective radiance for melanopsin (‘melanopic radiance’) to be modulated independent of chromaticity and luminance (Allen, 2018). Thus, by applying the principles of receptor silent substitution (Estévez & Spekreijse, 1982), it is possible to employ different combinations of more than 3 primaries to generate the same colour coordinate with differing melanopic radiance, these stimuli are termed ‘metameric’. This approach represents a unique opportunity to study melanopsin’s contribution to perception in human subjects.

Accurate colour rendering is a pre-requisite for a functional multiprimary display. Colour is typically described as a 3-dimensional (3D) coordinate derived from empirically defined colour matching functions and related to the activity of the three types of human cone photoreceptor (long-, medium-, and short- wavelength sensitive (LMS) cones). Numerous variants of such 3D colour spaces have been employed (e.g. RGB, LAB, XYZ (CIE, 2006)) but in each case, the appearance of a multispectral light is predicted by integrating its spectral power density with three spectral sensitivity functions. Central to this approach is the assumption that lights with equivalent colour coordinate should appear the same even if differing substantially in spectral composition (Estévez & Spekreijse, 1982). Accordingly, it is conceptually straightforward to produce light of a target colour coordinate using a multiprimary display by controlling the intensity of individual primaries.

Here we set out to generate full colour images using a five-primary (violet, cyan, green, yellow, red) display by recreating a target 3D colour coordinate for each pixel. The additional primaries allow many solutions to the problem of recreating a given 3D colour coordinate. Thus, our further aim was to exploit this opportunity by producing pairs of images with equivalent colour coordinates (known as ‘metamers’), but which either maximised or minimised output of the cyan primary (to enhance or reduce melanopic radiance, respectively).

One concern with using a narrow-band short-wavelength primary such as cyan, however, is the potential to perceive Maxwell’s spot. Maxwell’s spot is an entopic phenomenon that appears as a red spot in the central visual field. It occurs due to absorption of light by the macular pigment, which is most sensitive in the blue portion of the spectrum. Due to the macular pigment, two versions of colour matching functions exist: those that relate to the central (2°) visual field and those encompassing the periphery (10°). This means that colour coordinates can be metameric in the peripheral visual field, but may not be in the central, and this is evidenced by the appearance of Maxwell’s spot. This phenomenon was first reported when observing blue light (Maxwell, 1856) and can be simulated with the use of dichroic filters (Miles, 1954, Isobe and Motokawa, 1955). Maxwell’s spot has been reported in other systems utilising a short-wavelength primary (Palmer, 1978, Spitschan et al., 2017). Applications of receptor silent substitution to modulate melanopsin typically avoid such problems by blocking the central visual field (e.g. Barrionuevo and Cao, 2016, Spitschan et al., 2014, Tsujimura and Tokuda, 2011, Zele et al., 2018a) however, this is not a viable solution under conditions of free-viewing.

In our system, we indeed observe Maxwell’s spot exclusively in stimuli incorporating the cyan primary. To our knowledge, this is the first study to report Maxwell’s spot in spatially structured, full colour images. Here we set out to determine what spectral features induce Maxwell’s spot in some conditions, but not in others, and whether it is possible to eliminate this colour anomaly whilst still incorporating the cyan primary. We find that a uniform colour appearance can be achieved by changing the colour mapping used to render images, including a parameter that accounts for differences in colour coordinate between central and peripheral vision.

## 2 Methods

### 2.1 Experimental setup

The visual display used in this study is developed from one previously described (Allen, 2018). This modified five-primary display was created by superimposing images from two LCD projectors (Hitachi, model: CP-X5022WN) on a system utilising NVIDIA Quadro KS200 graphics card. Each projector had a 60 Hz refresh rate and the resolution of the projected display was 1280 × 1024 pixels. Superimposition was achieved by aligning the projected outputs using horizontal and vertical lens adjustments and then further refining by digital cornerstone correction using built-in software. Successful alignment was confirmed visually by the researcher using a variety of high spatial frequency stimuli. Each projector contained a white light source with dichroic filters producing distinct channels of red, green and blue. We inserted band-pass filters into the light path of these channels to produce 5 primaries of violet, cyan, green, yellow and red. The primaries were generated using the following filters: a 463–571 nm magenta notch filter (MidOpt, Palatine, IL; part# 102340892) in the blue channel to produce violet (xyY coordinate (0.154, 0.0499)), a 470 nm cut-off yellow longpass filter (PIXELTEQ, Largo, FL; part# LP470-r40x25x1) in the blue channel to generate cyan (0.0807, 0.3234), a 550 nm bandpass filter (PIXELTEQ, Largo, FL part # Bi550-r40x25x1) in the green channel to produce a narrower green (0.3077, 0.6430), a 463–571 nm notch filter (MidOpt, Palatine, IL; part# 102340892) in the green channel to make yellow (0.5189, 0.4572) and the red channel was left unmodified (0.6171, 0.3307). The maximum luminance of each channel was 19.62, 41.59, 389.0, 248.6 and 144.8 cd/m^2^ for violet, cyan, green, yellow and red respectively. A gamma correction was applied so that the range of greyscale values with and without the cyan primary were equivalent.

Presenting images using these primaries allowed up to ~3 fold difference in melanopsin excitation between stimuli utilising the cyan primary and stimuli that did not whilst retaining effective intensity for cones, as measured with standard measurement for visual displays (Commission Internationale de l’Eclairage (CIE) 10° Standard observer). Due to the relative similarity between the spectral sensitivities of melanopsin and rod opsin (peaks of 480 nm and 498 nm respectively) metameric stimuli were not rod-silent as this almost halves the melanopsin contrast achievable between metamers. To allow comparisons with RGB images an additional RGB LCD projector was used, this projector contained a 0.3 neutral density filter to place all projectors within a similar operating range. In other words, for a maximum luminance ‘white’ stimulus rendered with either display, at least one channel was set at its maximum 8-bit output (i.e. 255).

### 2.2 Calibration

All stimuli were generated using MATLAB version 9.2.0.556344 for Windows (The MathWorks, 2018). The output of each primary was measured independently in 8-bit colour range using a SpectroCAL spectroradiometer (Cambridge Research Systems) at regular intervals. Photon fluxes were calculated and linearly interpolated to obtain an input–output relationship for each channel. This was used to calculate the XYZ coordinates of each primary, which was then used to calculate intensities required to generate each coordinate. Successful calibration was confirmed by measuring output from projecting single colours from across the gamut (mean Euclidean difference in xy between target and presented colour coordinates = 0.0125 SD 0.004).

### 2.3 Stimuli generation

All stimuli in this study were generated using the principle of receptor silent substitution (Estévez & Spekreijse, 1982). Visual stimuli, termed “metamers” can be composed of varying combinations of primaries resulting in selective stimulation of a given class of photoreceptors (the ‘target’ class) whilst maintaining constant stimulation of the others (termed ‘silent’ classes). To achieve this one requires n + 1 available primaries, where n = the number of silent classes. In this study this principle was used to generate metameric stimuli with maximal difference in melanopsin excitation with LMS cones as silent classes. This was calculated using XYZ colour space (CIE, 2006). As melanopsin is maximally sensitive to 480 nm light (Berson et al., 2002) it is unsurprising that stimuli with high melanopsin activation contained a large contribution from the cyan primary (peak 485 nm), and corresponding metamers with low melanopsin excitation excluded this primary and had compensatory increases in the other primaries to achieve the same chromaticity. Not all primaries were used for all stimuli as the script calculated a parsimonious solution to achieving the same colour coordinate and in most cases this resulted in 3 or 4 primaries.

For images, a selection of RGB images were inputted and converted to CIE XYZ colour space for processing using available colour matching functions (CIE, 2005; Stockman & Sharpe, 2000). Verbal descriptions and chromaticity information for these images is available in Table S1. Metameric stimuli were generated that varied up to ~3 fold in their excitation of melanopsin and designed to be presented using the two projectors. Metameric versions of 19 images were presented to participants for them to rate based on colour appearance.

**Fig. 1:**
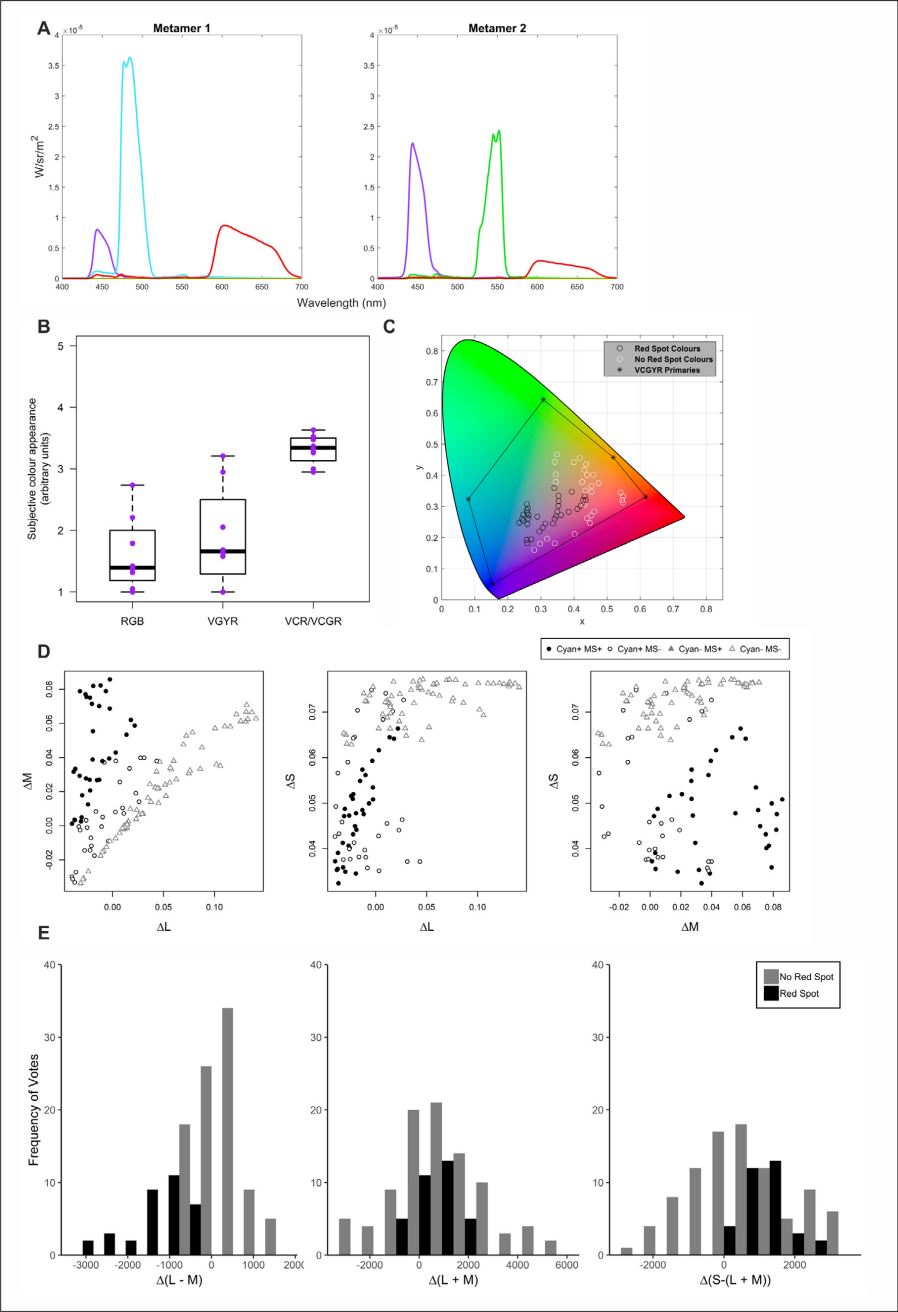
Colour anomaly in stimuli rendered with cyan primary correlates with differences in effective excitation of central and peripheral cones A) Our pentamic display can present metamers with varying primary contributions, shown here are example spectra for metamers, using the V, C and R or V, G and R primaries. B) Participants were shown an RGB reference image followed by test images comprising either another standard RGB image (control) or metameric images rendered with the pentamic display either with (VCR/VCGR) or without (VGYR) the cyan primary. Presented here are box-and-whisker plots showing the rating of image colour for each participant (purple dots) averaged across a total of 19 images. A low rating indicated natural and pleasant colours. Participants consistently rated images presented with the cyan primary as having unpleasant and unnatural colours. N = 8. Primaries are V = violet, C = cyan, G = green, Y = yellow and R = red. C) Chromaticity (in xyY colour space) of discs scored for appearance of reddish spot. Discs for which at least 4 of the 6 participants reported red spot in black. Black stars and connecting lines indicate the location of the five primaries and achievable gamut of the display. D) Differences in L (ΔL^10/2^), M (ΔM^10/2^) and S (ΔS^10/2^) coordinates between the 2° and 10° visual fields ((10°/2°) −1) for each disc; filled symbols indicate discs for which at least 4 of the 6 participants reported a red spot. Circles represent discs rendered with, and triangles without, the cyan primary. E) Histograms showing the frequency of reporting reddish spot (black bars) as a function of differences in cardinal axes of colour vision (Δ(L – M); Δ(L + M); Δ(S – (L + M))) between central and peripheral visual fields (10°–2°). N = 6 participants, 63 metameric pairs of stimuli. (For interpretation of the references to colour in this figure legend, the reader is referred to the web version of this article.)

For generating discs of uniform colour, colours were chosen from hyperspectral images of natural scenes (Fig. 1C, Fig. S1, Table S2) (Nascimento et al., 2002, Foster et al., 2006, Foster et al., 2016, Nascimento et al., 2016). A subset of colours were chosen that spanned the visual gamut and that had sufficiently high luminance. Additionally the luminance was normalised so that the maximum was in-line with the maximum output of the projectors. These colours were presented to participants as discs on a dark background (background luminance 4.27 cd/m^2^).

A selection of hyperspectral images were also used to predict the relationship between central (2°) and peripheral (10°) colour spaces across the colour gamut. To achieve this, the spectral information from 9 hyperspectral images was processed to calculate the natural difference between 2° and 10° XYZ colour coordinates (10°/2° − 1) for all pixels. While this information covered a good proportion of the colour gamut, unsurprisingly this was incomplete. Thus, to establish the natural ΔXYZ across the colour gamut, the natural ΔXYZ values were smoothed and interpolated 3-dimensionally. Those data were then used as reference values to calculate natural ΔXYZ values.

Stimuli were analysed in LMS and XYZ colour spaces. Differences between central (2°) and peripheral (10°) vision were calculated as a proportion (10°/2° − 1) and are referred to as ΔX ΔY etc. Differences compared to natural (i.e. what is expected for that particular colour based upon our hyperspectral image analysis) were calculated as a subtraction (observed (10°/2° − 1) – natural (10°/2° − 1)) and are referred to as ΔΔX, ΔΔY etc. For the generation of constrained images a subset of 9 images from the 19 used in the rating experiments were used. The constrained versions were generated using an algorithm that, for each pixel, solved metamers to an inputted maximum ΔΔY. If this threshold was not achievable for a given pixel then lower thresholds were tested (in steps of 0.01) until the colour was solved. Stimulus presentation was controlled using Processing v.3.3.1 for Windows (The Processing Foundation, 2018).

### 2.4 Participants & instruction

9 healthy participants (6 female) were recruited to take part in this study with median age bracket 30 – 34y. Not all participants completed all tasks, with 6 – 8 individuals for each experiment. Participants did not wear glasses or contact lenses and reported normal colour vision. Normal red-green colour vision was confirmed with Ishihara test for colour deficiency (Ishihara, 2004). Participants provided full, informed consent prior to participating. This study received ethical approval from the University of Manchester Research Ethics Committee. Participants were seated in a room with the projectors as the sole light source. They sat parallel with the projector which presented images on a wall-mounted screen. The projections occupied a visual angle of 41° × 32°. Participants viewed stimuli in free-viewing conditions without fixations. Responses to stimuli were recorded electronically using a keyboard.

When viewing images participants were asked to rate them based on how “pleasant and natural the colours appear” with a rating of 1 indicating natural and pleasant colours and a rating of 5 indicating the converse. The images and display type (RGB, multiprimary with cyan and multiprimary without cyan) were randomised in their order of presentation, this order was consistent across participants. Due to the positioning of the projectors away from line-of-sight it was not possible for participants to discern which display type they were viewing at any given time.

Prior to analysing the presence of Maxwell’s spot in discs of uniform colour participants underwent a practice trial with 10 discs, half of which contained Maxwell’s spot as determined by the researcher. Participants were encouraged to move their gaze to facilitate its identification and all were able to identify Maxwell’s spot in these discs. In the experimental session participants viewed 126 colours (63 metamer-pairs) singularly, in random order, and reported if they could identify a spot and its colour. To eliminate spots that were reported as after-effects only those reported as red/pink/purple were analysed with those reported as yellow/green/blue/white excluded.

When viewing images with constrained ΔΔY, participants were first presented with an image from a previous session, which they rated as having unnatural and unpleasant colours to remind them of the phenomenon before being presented with constrained images in a randomised order. They were asked to rate these images based on how “apparent and prominent” they found Maxwell’s spot, with a rating of 1 indicating no observable Maxwell’s spot and a rating of 5 indicating a clear and prominent spot.

### 2.5 *In silico* analysis

To determine the extent to which changing the spectral composition of our cyan primary affected central and peripheral (L – M) we conducted analysis *in silico* in which we replaced our primary with a hypothetical monochromatic blue, the other 4 primaries remained unchanged. This hypothetical primary had all power at one frequency and the analyses were repeated with this frequency ranging from 410 to 510 nm with a 5 nm resolution. We calculated the difference in (L – M) between centre and periphery (calculated as 10°–2°) for colours rendered with this hypothetical set of primaries. We conducted this both on colours where all participants reported no Maxwell’s spot present, and colours where the majority (at least 4/6) of participants reported the spot.

### 2.6 Graphs & statistical analysis

Graphs were created in R v. 3.5.1 for Windows (R Core Team, 2018,) and MATLAB, using the Computational Colour Science using MATLAB toolbox. Statistical tests and data handling were performed in R. For correlation the test used was Spearman’s coefficient of rank correlation.

## 3 Results

A five-primary display, allowing independent control over chromaticity, luminance, and melanopic radiance at single pixel resolution, was generated by superimposing images from two modified LCD projectors (as described in Allen et al., 2019). With this system, we can generate pairs of photometrically equivalent stimuli that differ in spectral composition (‘metamers’; Fig. 1A). We generated metameric sets of a bank of 19 colour images of everyday objects or scenes in which the chromaticity and luminance of each pixel was matched across representations. One element of each metameric image set was a conventional RGB version produced with standard projector to act as a control; two further representations were produced with the five-primary display and rendered with the output of the cyan primary either maximised (to enhance melanopic radiance) or minimised (to reduce melanopic radiance). To determine whether all 3 representations were functional colour images, we asked healthy human volunteers to rate the acceptability of colour reproduction. We found that while RGB and non-cyan-primary versions of the images were approximately equally acceptable, images rendered with high output of the cyan primary had lower satisfaction ratings (Fig. 1B). In the images rendered with the cyan primary, participants frequently reported the appearance of a red or pink spot that travelled with gaze, to lie always in the centre of the visual field. This description, and the subjective experience of the authors, matched that of the Maxwell’s spot phenomenon caused by macula pigment-dependent absorbance of shorter wavelength light in the central retina. The spot itself had a concentrated centre, with intensity fading towards the periphery, similar to previous reports (Miles, 1954).

To catalogue this colour anomaly more systematically, we analysed its occurrence in large coloured discs (diameter = 15° of visual space) presented with the pentamic display. Stimuli spanned the visual gamut and were constructed as metamer pairs either maximising or minimising use of the cyan primary. The colour anomaly reported in colour images was also apparent with these uniform discs, with all participants reporting a pink/red spot in the central visual field for at least one colour, and at least 4 of the 6 participants doing so in 34 out of the 126 stimuli presented (Fig. 1C). Appearance of the red spot was restricted to stimuli generated using the cyan primary (Fig. 1D).

If the reported red spot was indeed a manifestation of Maxwell’s spot, its appearance should have correlated with differences in the predicted colour between central and peripheral retina. To explore this possibility, we used estimates of cone spectral sensitivity for central and peripheral visual fields to calculate location of each stimulus in 2° and 10° LMS colour space. We found that stimuli inducing the red spot percept formed a distinct cluster when plotted as a function of differences in 2° vs 10° LMS coordinates (Fig. 1D). Specifically, a spot was identified most often in discs with a large difference in M coordinate between central and peripheral vision (ΔM) and a small difference in L (ΔL) (Fig. 1D). ΔS was generally rather small for these stimuli and was not correlated with spot appearance.

Analysing the data in terms of cone-opponent mechanisms reveals that the appearance of this colour anomaly correlates with differences in the (L – M) (red-green) axis between centre and periphery (Fig. 1E). Thus, the spot was reported in conditions in which Δ(L – M) (peripheral-central) was low (Fig. 1E). These data support the conclusion that the anomaly reflects differences in central vs peripheral spectral sensitivities and is a manifestation of the Maxwell’s spot phenomenon.

**Fig. 2:**
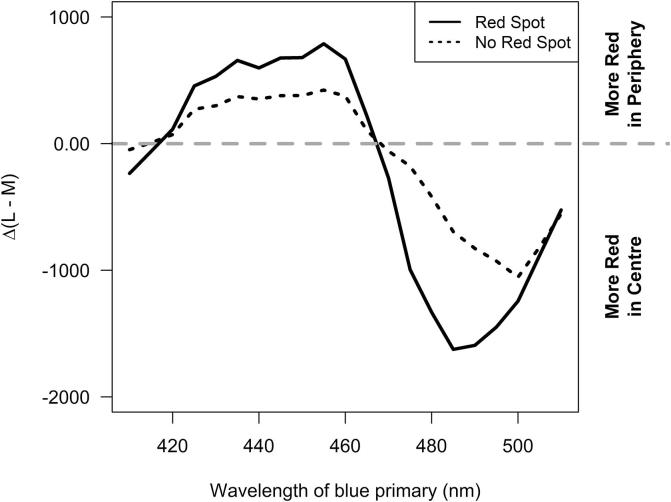
Inclusion of a primary across the cyan range is predicted to impact Δ(L-M) between central and peripheral vision. Simulated Δ(L – M) (as 10°–2°) for coloured discs rendered using adaptations of the pentamic display design in which the cyan primary was replaced by a hypothetical monochromatic primary across the 410–510 nm wavelength range. Δ(L – M) is reported as median for all colours, black solid line for those colours eliciting Maxwell's spot (as determined by at least 4/6 participants when rendered with our actual pentamic display); black dashed line for those which did not for any participants.

Given that the Maxwell’s spot was only reported in stimuli that contained significant output from our cyan primary, we sought to determine the extent to which changing the spectral composition of this primary within our system (i.e. all other primaries held constant) could influence Δ(L – M). We achieved this *in silico* by calculating the effect of replacing our cyan primary with a hypothetical monochromatic primary at wavelengths spanning 410–510 nm (while fixing the spectral composition of the remaining 4 primaries) on Δ(L – M) for a range of colours. We found that this manipulation had a systematic impact on Δ(L – M), with this parameter being more negative when primaries in the 470–510 nm range were employed (Fig. 2). To relate this to the appearance of Maxwell spot, we plotted Δ(L – M) as a function of ‘cyan’ primary wavelength separately for those colours in which we had observed a Maxwell’s spot using our five-primary display (Fig. 2). As may have been predicted, Δ(L – M) was particularly low in these colours and for primaries in the ~470–510 nm range (corresponding to the peak output of our original cyan primary (475–490 nm); Fig. 1A).

**Fig. 3:**
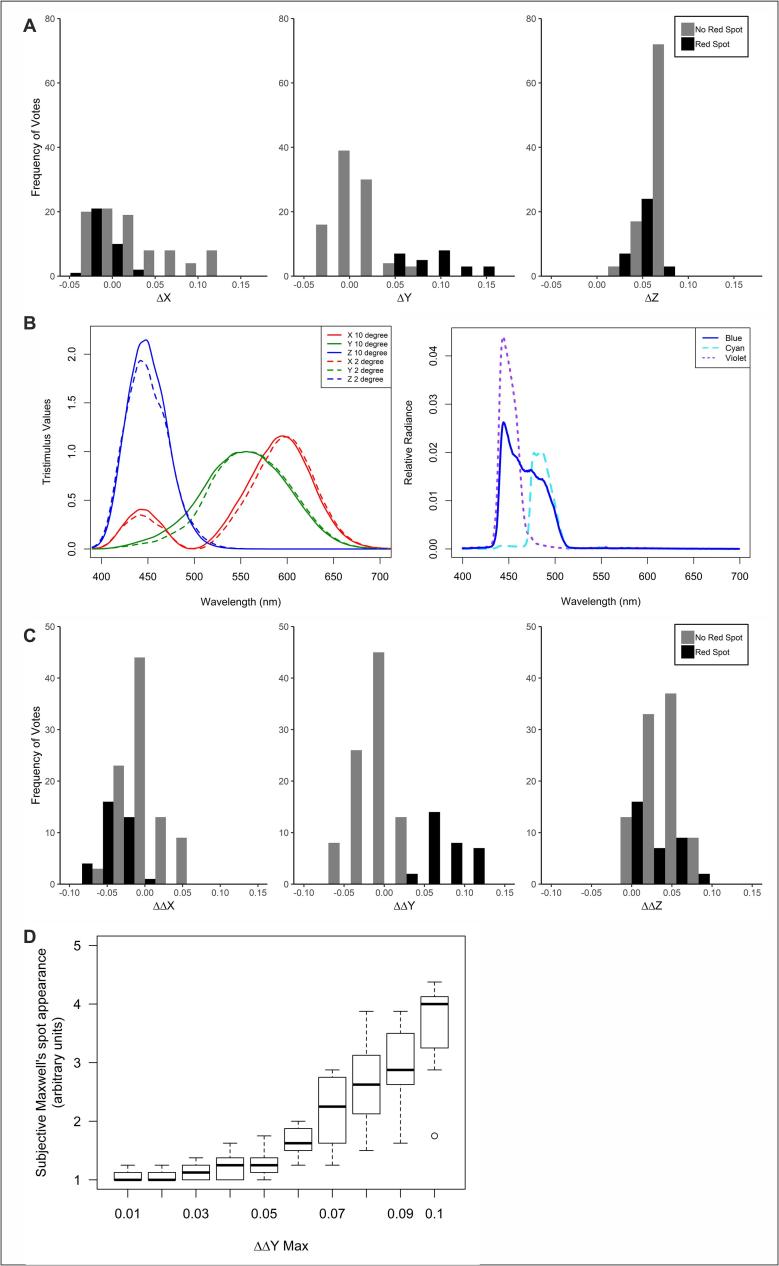
Appearance of Maxwell’s spot correlates with the degree of divergence from naturally occurring differences in central vs peripheral colour. A) Histograms showing the distribution of colours that did (black) or did not (grey) elicit Maxwell’s spot (data replotted from Fig. 2) as a function of the difference in central vs peripheral colour coordinate in XYZ colour space (ΔX, ΔY and ΔZ, calculated as (10°/2° − 1)). Maxwell’s spot positive colours are determined by a positive rating from at least 4/6 participants B) The XYZ colour matching functions plotted for 10° (solid lines) and 2° (dashed lines) visual fields alongside the spectral power output of Violet and Cyan primaries from our pentamic display, and the Blue from a standard RGB display, normalised for area under the curve. C) distributions of the data in panel A as a function of the divergence between the difference in colour coordinate (10°/2° − 1) when rendered with the pentamic display vs that observed for that colour in hyperspectral images of natural scenes (ΔΔX, ΔΔY and ΔΔZ). D) The impact on subjective appearance of Maxwell’s spot of constraining maximum allowable ΔΔY for each pixel when rendering images using the pentamic display. Data show subjective Maxwell’s spot appearance (median = 1; range = 4; higher ratings indicating prominent Maxwell’s spot) for a bank of 9 everyday images as a function of the maximum allowable ΔΔY. The just noticeable difference for Maxwell’s spot detection is ΔΔY = 0.06. N = 8 participants. (For interpretation of the references to colour in this figure legend, the reader is referred to the web version of this article.)

These data raise the possibility of eliminating Maxwell’s spot in pentamic images by including a suitable constraint in image processing: to place a ceiling on Δ(L – M). To explore how this could be implemented in practice, we moved to considering the location of our coloured disc stimuli in XYZ colour space (the availability of both 10 and 2° colour matching functions in this space facilitates image manipulation). XYZ is a linear transformation from LMS and, as expected, the appearance of Maxwell’s spot in our stimuli correlated with differences in coordinates in 10 and 2° XYZ space (Fig. 3A). The clearest distinction between stimuli inducing Maxwell’s spot and those that did not was the difference in the Y coordinate in 10 vs 2° colour space. This is to be expected as differences between centre and periphery are largest in this parameter across the range where our cyan primary is active (Fig. 3B).

The correlation with ΔY implies that minimising this parameter in image generation could reduce the appearance of Maxwell’s spot. However, differences in central and peripheral colour coordinates also occur in nature, yet we do not experience Maxwell’s spot in such conditions. We therefore reasoned that the appearance of Maxwell’s spot might be more appropriately considered in terms of how our artificial stimuli differed from those encountered in nature. There are additional practical reasons for using this approach in that, some colours naturally have a large ΔY and can therefore not be constrained to a small value. Attempting to do so would reduce the presentable colour gamut.

To calculate ‘expected’ colour differences between central and peripheral vision we turned to a bank of hyperspectral images. We identified pixels in these images whose colour matched that of the bank of test colours used to create the discs that had been scored for appearance of Maxwell’s spot. Calculating the difference in central vs peripheral colour coordinate for these natural pixels enabled us to estimate the divergence in these coordinates between our artificial stimulus and a natural element of equivalent colour (defined as ΔΔX, ΔΔY and ΔΔZ). We found that indeed discs inducing Maxwell’s spot can be distinguished from those that do not on the basis of their central and peripheral differences in XYZ compared to those found in hyperspectral images. In particular, Maxwell’s spot correlated closely with ΔΔY (Fig. 3C, Spearman’s r = 0.80, p < 0.001 with average participant rating), appearing only when differences in the Y coordinate between centre and periphery were larger than those found in natural images (ΔΔY > 0).

The correlation between the appearance of Maxwell’s spot and high values of ΔΔY raise the possibility that constraining this parameter could provide a relatively simple approach to eliminating Maxwell’s spot from images presented with the pentamic display. To test this we applied an additional ceiling constraint on ΔΔY for each pixel when rendering colour images for presentation on our pentamic display. Note that in each image only that minority of pixels whose ΔΔY would in any case be above the threshold were impacted. ΔΔX and ΔΔZ were left uncontrolled and clamping ΔΔY produced concomitant changes in these (Fig. S2). When presenting these constrained images to participants there was a significant positive correlation between the maximum allowable ΔΔY and the likelihood of reporting Maxwell’s spot (Spearman’s r = 0.69, p < 0.001, Fig. 3D). The just noticeable difference for ΔΔY and reporting of Maxwell’s spot was 0.06, with 58% of observations at this constraint reporting the phenomenon (a rating of 2 or higher, Fig. 3D). These data confirm that constraining ΔΔY is a viable approach to eliminating Maxwell’s spot in stimuli presented with the pentamic display.

## 4 Discussion

We report here the appearance of a colour anomaly when rendering colours using a multiprimary display that includes a cyan primary. This anomaly has hallmarks of Maxwell’s spot, in that it occupies the central field of view irrespective of direction of gaze, appears reddish with colour fading from its centre to periphery, and is associated with differences in colour coordinate between central and peripheral vision (Fig. 1) (Miles, 1954). The anomaly is also sufficiently intrusive as to impair the appearance of colour images produced using the cyan primary. To address this issue, we have identified a pragmatic approach to avoiding this anomaly, whereby we introduce an additional constraint in colour processing, in which the difference in the Y dimension between 2° and 10° versions of XYZ colour space is held close to that encountered in nature (Fig. 3).

Previous studies have reported Maxwell’s spot when using narrow-band blue light (Maxwell, 1856, Miles, 1954, Isobe and Motokawa, 1955). In line with these findings our *in silico* analysis suggests that any system employing a cyan primary could encounter this phenomenon. Thus, we find that the low values for Δ(L – M), which correlate with Maxwell’s spot appearance in our dataset (Fig. 1E), are predicted when using narrowband primaries across the 470–500 nm range (Fig. 2). This is in accordance with other studies utilising displays containing narrowband blue primaries, which also report this phenomenon (Palmer, 1978, Spitschan et al., 2017). To our knowledge, however, the present study is the first to show Maxwell’s spot in spatially structured stimuli. Our *in silico* analysis indicates that using broader band primaries or narrowband primaries at shorter wavelengths could alleviate this problem, and likely explain why Maxwell spot is infrequently encountered in conventional RGB displays. However, these strategies minimise the degree to which such a display could be used to control effective radiance for melanopsin (whose spectral sensitivity peaks at this portion of the spectrum).

One potential solution to eliminating Maxwell’s spot in circumstances in which a fairly narrow band cyan primary is required is to apply an additional step in image processing to constrain the difference in central vs peripheral colour coordinate. Our investigation of the correlation between Maxwell’s spot appearance and differences in central and peripheral colour spaces reveals that a great deal of the effect can be predicted based upon the Y coordinate of XYZ space (Fig. 3). Thus, the appearance of Maxwell’s spot correlates with high values for the difference between 2° vs 10° versions of the Y coordinate (ΔY). Although this is an empirical observation, one can generate a relatively simple post hoc explanation based upon the spectral sensitivity functions of the X, Y and Z functions. Thus, differences between 2 and 10° sensitivity functions are greatest for the Y parameter across the wavelengths at which our cyan primary has strong output (470–490 nm) (Fig. 3B).

Having identified high ΔY as a potential origin for Maxwell’s spot we show that indeed constraining this parameter can reduce appearance of this colour anomaly (Fig. 3). Our approach was not to simply minimise ΔY but rather to minimise the difference between ΔY and that experienced in nature for that colour (designated ΔΔY). A practical reason for applying this approach is that some colours simply have a large ΔY that would be challenging to eliminate with any set of primaries. Due to the luteal pigment, most naturally encountered spectra should produce a difference in colour coordinates between centre and periphery. As we don't experience Maxwell’s spot in everyday viewing, a compensatory mechanism must address this expected difference. To account for this process, we used a bank of 9 hyperspectral images to calculate expected ΔY for a range of colours and, from that, the divergence in ΔY for these colours when presented on the five-primary display (ΔΔY). As expected, the appearance of Maxwell’s spot correlated with ΔΔY, and furthermore, constraining this parameter proved an effective strategy to generate acceptable colour images using the five-primary display (Fig. 3D).

A limitation of our approach is that the 2 and 10° colour matching functions used in this study are based on the standard observer, whereas in reality these vary between individuals and thus metamers based on this are imperfect for different participants. This could lead to inter-individual variations in image appearance. In the case of experimental applications of this approach to generate analytical stimuli, additional calibration steps to account for individual differences may be included (as has been done previously (Allen et al., 2019)). A further feature of the approach is that efforts to introduce variations in melanopic radiance will also produce contrast for rods (whose spectral sensitivity is similar to that of melanopsin) that could impact image appearance when viewing at lower radiances (Ikeda and Shimozono, 1981, Buck et al., 1998, Sun et al., 2001, Cao et al., 2008). Arguably, differences in rod excitation between centre and periphery could also be problematic when viewing a display at lower light levels. However, we find that applying the ΔΔY constraint is sufficient to also control any difference in central vs. peripheral rod excitation (data not shown).

One concern in applying the ΔΔY constraint is that this might preclude our fundamental purpose in introducing the cyan primary – to gain control of melanopsin. This does happen to a certain extent, as constricting ΔΔY does reduce the range of achievable melanopsin excitation (Fig. S3). For this application a compromise between minimising Maxwell’s spot and maximising the range of melanopic radiances may be required. Fortunately, we find that reducing Maxwell’s spot is achievable with relatively modest constraints in ΔΔY (Fig. 3D), which should produce small restrictions in melanopic radiance (Fig. S3).
