# Supplementary material for: Appearance of Maxwell’s spot in images rendered using a cyan primary
Source: Vision Res. 2019 Dec;165:72–9. doi: 10.1016/j.visres.2019.10.004 (PMC6902267; doi:10.1016/j.visres.2019.10.004)
Supplement: Supplementary data 1 [file mmc1.docx]

**Supplementary Data**

**Supplementary Figures S1-S3**


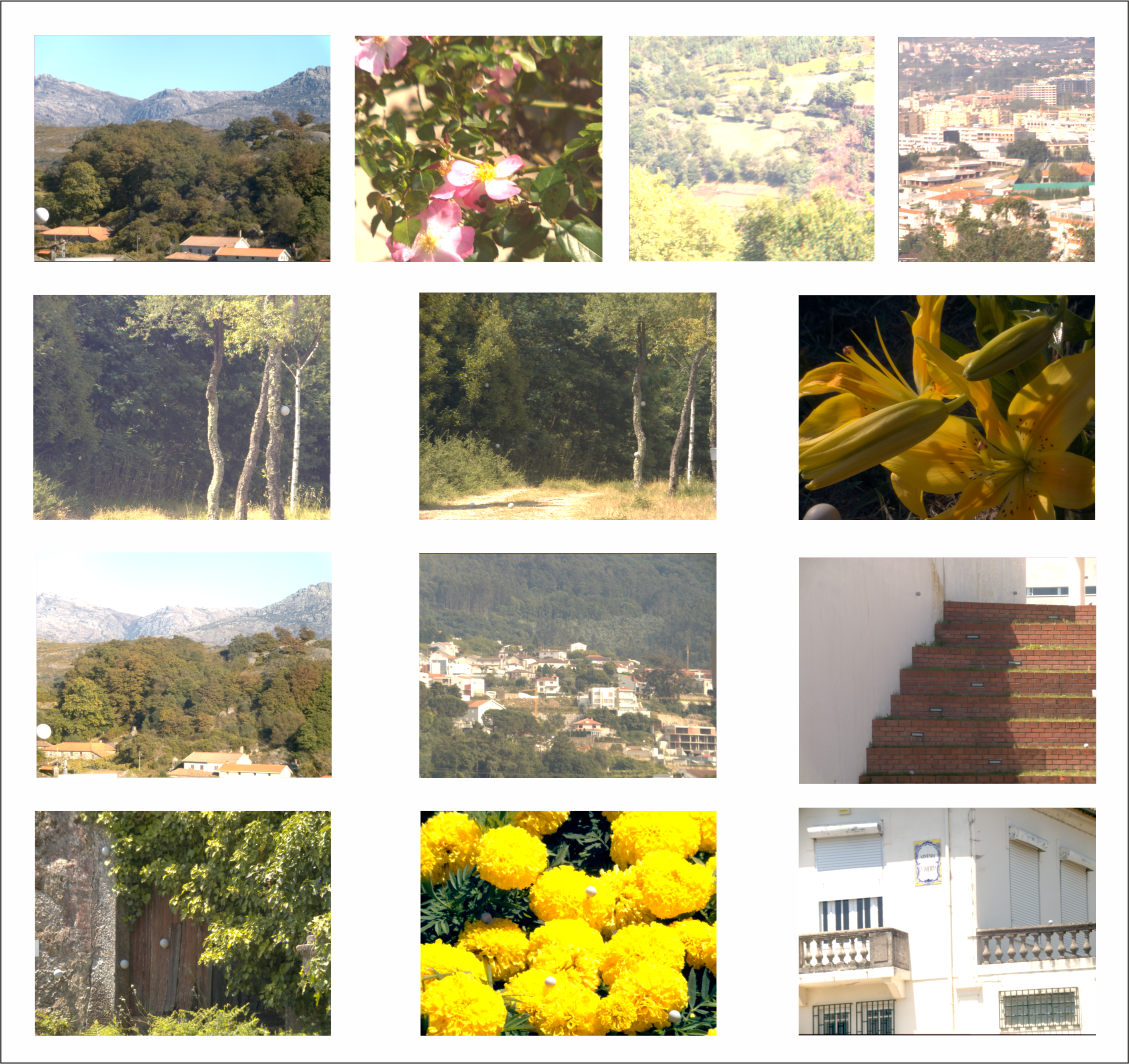


**Figure S1**. Hyperspectral images from which colours used in experiments were sampled. Images from Nascimento, Ferreira & Foster (2002); Foster et al., (2006); Foster, Amano & Nascimento, (2016); Nascimento, Amano & Foster, (2016).


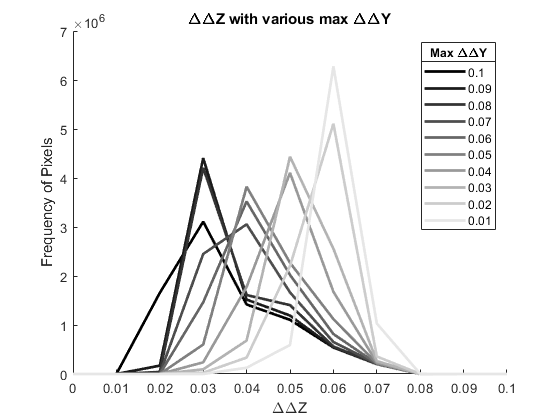

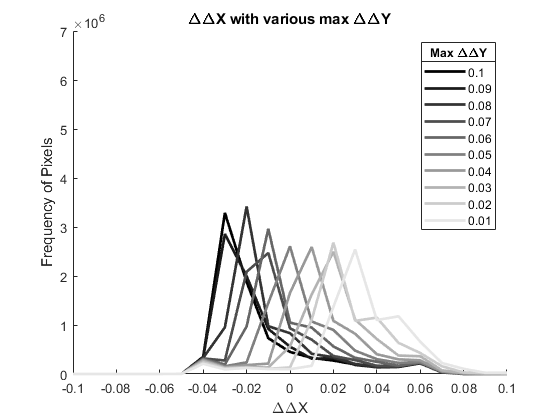


**A B**

**Figure S2** We generated images with maximum threshold ΔΔY for each pixel varying between 0.01-0.1 Due to the covariance of the XYZ parameters this also resulted in differences in ΔΔX (**A**) and ΔΔZ (**B**) across pixels.


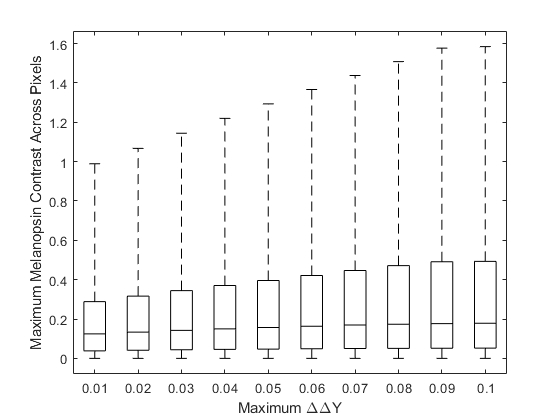


**Figure S3.** We generated images with maximum threshold ΔΔY varying between 0.01-0.1. We calculated the Michelson Contrast between lowest and highest achievable melanopsin excitation for each pixel across 9 images. Pixels across these images varied in their melanopsin excitation with tighter ΔΔY constraints resulting in reduced achievable contrast.

**Supplementary Tables S1 and S2**

**Table S1**. Luminance and chromaticity of images presented to participants, averaged across all pixels, alongside verbal description of image contents.

| x | y | *Y* | Image Description |
| --- | --- | --- | --- |
| 0.317798 | 0.33258 | 880.6468 | Brown mountain with white clouds |
| 0.359391 | 0.397175 | 856.4119 | Lake in green valley |
| 0.289478 | 0.299265 | 851.6147 | Cliff face amongst a green forest |
| 0.280962 | 0.30221 | 900.2605 | White owl against a dark background |
| 0.30037 | 0.33417 | 882.0355 | Berries against a grey background |
| 0.276377 | 0.333207 | 839.448 | Wooden path along sea and beach |
| 0.47345 | 0.457397 | 973.6845 | Red butterfly against flowers and leaves |
| 0.576553 | 0.354384 | 839.0827 | Red flowers against white and blue sky |
| 0.364811 | 0.311863 | 982.1542 | Red ladybird against green foliage |
| 0.254753 | 0.237427 | 765.5171 | Dragonfly against green foliage |
| 0.298802 | 0.281803 | 804.9506 | Sand and water against dark mountains |
| 0.334336 | 0.34289 | 834.2593 | Dark tree against a yellow sunset |
| 0.285576 | 0.337228 | 854.9506 | Green rice fields |
| 0.312727 | 0.329023 | 832.2785 | Yellow peppers |
| 0.35505 | 0.307442 | 841.577 | Orange autumn leaves |
| 0.432788 | 0.45806 | 857.4811 | Berries and cake against a pink background |
| 0.359002 | 0.278438 | 896.4586 | Tropical fish against a blue background |
| 0.388721 | 0.463086 | 882.3643 | A clownfish against light coral |
| 0.382529 | 0.49297 | 986.4605 | A hummingbird against a grey background |

**Table S2**. xyY coordinates of all stimuli presented to participants as discs of uniform colour, those in which at least 6/9 participants identified Maxwell’s spot are depicted in bold font (values plotted in Fig 2a).

| *x* | *y* | Y |
| --- | --- | --- |
| **0.258583** | **0.181905** | **694.3059** |
| **0.349526** | **0.283214** | **715.3685** |
| **0.235018** | **0.247658** | **688.3729** |
| **0.341656** | **0.26034** | **686.2796** |
| **0.257726** | **0.307934** | **689.785** |
| **0.243262** | **0.26018** | **717.5429** |
| **0.289125** | **0.26709** | **763.5223** |
| **0.246664** | **0.273592** | **729.9343** |
| **0.420547** | **0.302189** | **682.423** |
| **0.346241** | **0.268857** | **775.4282** |
| **0.409916** | **0.291637** | **720.4326** |
| **0.25672** | **0.192699** | **690.9125** |
| **0.432003** | **0.332722** | **702.0843** |
| **0.35333** | **0.334241** | **710.2975** |
| **0.258572** | **0.279129** | **731.9864** |
| **0.427631** | **0.319789** | **687.1006** |
| **0.305916** | **0.231784** | **694.0949** |
| **0.260267** | **0.293855** | **689.202** |
| **0.340479** | **0.359185** | **690.5757** |
| **0.257246** | **0.287206** | **749.3017** |
| **0.33641** | **0.244914** | **764.6608** |
| **0.246273** | **0.254059** | **688.0814** |
| **0.435366** | **0.320256** | **727.8823** |
| **0.295209** | **0.219511** | **686.1341** |
| **0.384143** | **0.282457** | **683.0336** |
| **0.353158** | **0.301735** | **705.9385** |
| **0.259282** | **0.242897** | **686.7055** |
| **0.313784** | **0.244853** | **710.7915** |
| **0.262012** | **0.261098** | **711.0333** |
| **0.270727** | **0.194732** | **714.9791** |
| **0.353766** | **0.317181** | **708.3541** |
| **0.263111** | **0.272326** | **760.6925** |
| **0.393116** | **0.347048** | **700.4067** |
| **0.360913** | **0.272425** | **705.7128** |
| 0.458484 | 0.401726 | 774.6914 |
| 0.45632 | 0.291214 | 697.396 |
| 0.279989 | 0.160398 | 683.4663 |
| 0.337329 | 0.455416 | 732.5933 |
| 0.25321 | 0.343431 | 701.1434 |
| 0.307849 | 0.266489 | 721.244 |
| 0.445569 | 0.38044 | 731.3382 |
| 0.297536 | 0.180115 | 695.0658 |
| 0.332984 | 0.266125 | 778.5971 |
| 0.474237 | 0.374504 | 716.072 |
| 0.268406 | 0.214039 | 725.2582 |
| 0.344909 | 0.329681 | 717.276 |
| 0.417133 | 0.45556 | 719.8087 |
| 0.402248 | 0.211215 | 703.9247 |
| 0.403784 | 0.311038 | 729.8237 |
| 0.344843 | 0.306785 | 726.634 |
| 0.447606 | 0.363028 | 709.5474 |
| 0.547252 | 0.338355 | 735.3179 |
| 0.279372 | 0.166376 | 685.633 |
| 0.313969 | 0.205999 | 724.6802 |
| 0.294539 | 0.18806 | 698.3935 |
| 0.453379 | 0.346749 | 704.5492 |
| 0.468678 | 0.385734 | 720.7571 |
| 0.241194 | 0.295976 | 735.0672 |
| 0.396725 | 0.449001 | 703.6216 |
| 0.257458 | 0.29132 | 725.1237 |
| 0.2534 | 0.313539 | 749.6342 |
| 0.433324 | 0.408902 | 774.5283 |
| 0.424803 | 0.378547 | 709.048 |
| 0.461203 | 0.283721 | 695.5396 |
| 0.337925 | 0.417444 | 723.2888 |
| 0.447973 | 0.24676 | 728.7393 |
| 0.258806 | 0.30468 | 784.1047 |
| 0.543278 | 0.325325 | 720.7976 |
| 0.54793 | 0.332914 | 733.4339 |
| 0.303287 | 0.253976 | 701.6421 |
| 0.40157 | 0.211566 | 704.7005 |
| 0.418017 | 0.33853 | 693.1572 |
| 0.41526 | 0.462223 | 721.5722 |
| 0.418135 | 0.390475 | 714.0366 |
| 0.243322 | 0.283959 | 699.027 |
| 0.452792 | 0.365668 | 725.8647 |
| 0.54897 | 0.317795 | 726.9624 |
| 0.414299 | 0.31673 | 686.3435 |
| 0.337089 | 0.279909 | 691.9636 |
| 0.431294 | 0.417519 | 713.4667 |
| 0.242749 | 0.309823 | 750.0909 |
| 0.439956 | 0.262084 | 698.2762 |
| 0.345051 | 0.378747 | 691.4897 |
| 0.439662 | 0.400794 | 767.7859 |
| 0.431509 | 0.30572 | 690.6397 |
| 0.25557 | 0.330216 | 701.2253 |
| 0.536145 | 0.351552 | 742.8345 |
| 0.291696 | 0.239458 | 692.1104 |
| 0.446134 | 0.248665 | 729.6177 |
| 0.399238 | 0.442005 | 702.2404 |
| 0.453967 | 0.280474 | 680.9968 |
| 0.436974 | 0.267352 | 701.3087 |
| 0.284118 | 0.297951 | 786.2279 |
| 0.45292 | 0.408526 | 781.6235 |
| 0.345025 | 0.40278 | 717.0186 |
| 0.340834 | 0.183532 | 718.8578 |
| 0.341849 | 0.181691 | 717.4863 |
| 0.343734 | 0.36234 | 722.3208 |
| 0.42882 | 0.365493 | 683.4617 |
| 0.452331 | 0.270957 | 679.4162 |
| 0.257846 | 0.272402 | 696.7824 |
| 0.356437 | 0.292043 | 713.6535 |
| 0.341577 | 0.443159 | 725.7512 |
| 0.232919 | 0.279494 | 699.0455 |
| 0.344437 | 0.479001 | 782.9719 |
| 0.341543 | 0.29291 | 794.5235 |
| 0.341036 | 0.395692 | 696.5988 |
| 0.385782 | 0.369317 | 707.9442 |
| 0.546103 | 0.320896 | 718.5409 |
| 0.333373 | 0.381528 | 696.9455 |
| 0.541019 | 0.344282 | 738.9349 |
| 0.348789 | 0.466584 | 776.3098 |
| 0.43428 | 0.350852 | 680.227 |
| 0.345294 | 0.345657 | 720.0948 |
| 0.257002 | 0.19723 | 701.7342 |
| 0.431807 | 0.438107 | 723.3225 |
| 0.251768 | 0.322834 | 771.1527 |
| 0.424997 | 0.3387 | 738.1421 |
| 0.436777 | 0.435047 | 720.5006 |
| 0.379488 | 0.299147 | 687.7396 |
| 0.425013 | 0.352223 | 708.5537 |
| 0.422652 | 0.32438 | 696.2032 |
| 0.255276 | 0.210584 | 698.0677 |
| 0.547513 | 0.312098 | 724.7891 |
| 0.318629 | 0.196324 | 719.1234 |
| 0.427536 | 0.424806 | 715.916 |
